# The Scientific Biography of P.Ya. Galperin: Stages of Life and Creative Work

**DOI:** 10.11621/pir.2022.0401

**Published:** 2022-12-15

**Authors:** Marina A. Stepanova

**Affiliations:** a Lomonosov Moscow State University, Moscow, Russia

**Keywords:** History of psychology, Lomonosov Moscow State University, MGU, scientific biography of P.Ya. Galperin, P.Ya. Galperin’s theory of stage-by-stage formation of mental actions and concepts, general psychological theory of activity, non-classical psychology

## Abstract

**Background:**

This article is dedicated to the 120th anniversary of the birth of Piotr Yakovlevich Galperin (1902–1988), an outstanding Soviet psychologist, the author of an original psychological concept and scientific school, and an organizer of psychological science.

**Objective:**

To reconstruct the main stages of the scientific biography of Piotr Yakovlevich Galperin.

**Results:**

The paper demonstrates the internal logic of P.Ya. Galperin’s developing scientific views in creating the theory of stage-by-stage formation of mental actions and concepts, which analyzes the process of formation of the main components of mental activity and develops a system of conditions for transforming an objective action into a psychological phenomenon.

This biography is based on Galperin’s publications and speeches, memoirs of associates and family members, and numerous archival materials.

All the periods of Galperin’s life are presented, reflecting his participation, starting from the mid-1920s, in scientific and scientific-practical events. Particular attention is paid to Galperin’s work at M.V. Lomonosov Moscow State University (MGU): 55 years of Galperin’s professional and personal life (from 1943 until his death in 1988) were associated with the Philosophy Faculty, and then with the Psychology Faculty.

**Conclusion:**

The importance of preserving P.Ya. Galperin’s scientific legacy is shown and steps taken in this direction are indicated.

*The greatest use of a life is to spend it for something that outlasts it*.

*W. James*

This year, 2022, marks the 120-year jubilee of Piotr Yakovlevich Galperin (October 2, 1902 — March 25, 1988) and the 70th anniversary of the first official mention of a new psychological approach, the orienting function of the psyche as a subject of psychological research. These anniversaries are a worthy occasion to look at Galperin’s scientific legacy, to recognize his philosophical and psychological ideas and their subsequent influence on science and practice, as well as the facts of his scientific biography, which reflects milestones in the development of Soviet psychological thought.

On the 90th anniversary of Galperin’s birth, V.P. Zinchenko wrote an article in the journal *Voprosy psikhologii* [Questions of Psychology] titled “A word about a Teacher”: he acknowledged with regret that a scientific biography of P.Ya. Galperin had not yet been written. Zinchenko noted: “I cannot claim to describe it, since I was not his direct student, although I consider him my teacher” (Zinchenko, 1993, p. 89). Three decades later, on the 120th anniversary of Galperin’s birth, we are only approaching a solution to this difficult task, and the compilation of a complete scientific biography of Galperin, encompassing all periods of his life equally, is still a matter for the future. Not all the archives have yet been sorted out, and not all the research performed under Galperin’s direction has been carefully analyzed.

A brief essay about Galperin, titled “A teacher from the galaxy of sages,” was written by L.F. Obukhova for the book *Outstanding psychologists of Moscow* (Obukhova, 2007), which has gone through two editions. Information about the stages of Galperin’s creative work can be found in articles by L.I. Aidarova (2002), A.N. Zhdan (2007, 2017, 2018), V.P. Zinchenko (1993, 2021; Stil’ myshleniia…, 2011), N.N. Nechaev (2003, 2012), A.I. Podolskii (2002, 2007, 2017), A.N. Sidneva (2019), M.A. Stepanova (2012, 2016, 2020), and others.

We should note that a scientific biography is inseparable from a personal one, and this is true not only for Galperin. An example is the recently published biography of N.A. Bernshtein by I.E. Sirotkina (2021). A.R. Luria, in his scientific autobiography *Stages of the road traveled*, wrote that “in this book there is no hero with exceptional abilities; there is neither specific talent nor tragedy. But there is an atmosphere of real life…” (Luria, 1982, p. 181). Biographers of A.N. Leontiev quote him as saying: “A scientific autobiography cannot, it seems to me, be limited to the official list of completed works. Personally, I have always been more interested in *subjective aspects* of the descriptions of scientists’ lives: *how the scientist came to science*; the inner motives of his scientific life; how *he himself perceived* its events and what subjectively acted upon him — at one stage or another — as a ‘discovery’ made by him “ (emphasis added — M.S.) (Leontiev et al., 2005, p. 8). The outstanding Russian thinker D. Merezhkovskii (2007) called attention to the importance of a subjective view of a writer’s life, a point which is also true of a scientist.

It is this approach that seems the most productive, and it formed the basis of P.Ya. Galperin’s scientific biography.

## Family and Education

P.Ya. Galperin was born on October 2 (new style), 1902 in Tambov. His father, Iakov Abramovich Galperin, was a district doctor at that time, and later became a well-known neurosurgeon and otolaryngologist. In 1911, the family moved to Kharkov, where his father taught at the Medical Institute and in 1930 became a professor in the Ear, Nose, and Throat Department; he died in 1937 (according to some sources, in 1938). P.Ya. Galperin’s daughter Sof ’ia Petrovna wrote in her memoirs that Piotr Yakovlevich tried all his life to imitate his father, and for years developed the habit of restraining himself and making compromises.

His mother, Sof ’ia Moiseevna Galperina (née Margulis), was a housewife; she died in 1917. After their mother’s death, there were three children left in the family: two sons (Piotr Yakovlevich, born in 1902; Teodor Yakovlevich, born in 1904) and a daughter (Polina Iakovlevna, born in 1908). Anna Ivanovna, an employee of Ia.A. Galperin, became their stepmother; she treated the children as if they were her own, but herself bore no children. Anna Ivanovna outlived her husband and died in 1961; she was buried in Kharkov next to Iakov Abramovich Galperin.

P.Ya. Galperin attended the classical gymnasium of the Society of Working Women in Kharkov, at which boys and girls were taught together. One of Galperin’s classmates was Tamara Izrailevna Meerzon, who later became his wife. He dedicated to her his only book published during his lifetime, *Introduction to psychology:* “To my dear friend, my wife, Tamara Izrailevna Meerzon.” In the gymnasium there were various groups for the study of socio-political and social sciences. Galperin attended a philosophical group led by Professor Stolpner, a translator of Hegel’s works into Russian. We can assume that this had an impact on the formation of the professional views and attitudes of the future psychologist.

In his youth, Galperin read books on philosophy and psychology that were in his father’s library, which even then aroused in him a desire to search for a method of objective study of human thinking. An attempt to implement this idea subsequently resulted in the study of optical illusions, on which Galperin prepared a short article for the German psychological journal *Zeitschrift für Psychologie* (Galperin, 1931). Later Galperin recognized the scientific limitations of that first study, but he did not abandon the search for an objective approach to mental life.

On the emphatic recommendation of his father, who advised him not to choose philosophy or psychology for his profession, P.Ya. Galperin followed his father’s example and from 1921 to 1926 studied at the Kharkov Medical Institute, specializing in psychoneurology. In 1924, while a student, he began scientific work at the Department of Nervous Diseases. Under the influence of Professor K.I. Platonov, who used hypnosis to treat neuroses and instead of anesthesia during operations, Gal-perin began to study the effect of hypnosis on digestive leukocytes. The results of the study formed the basis of Galperin’s first scientific publication (with P.P. Istomin) in 1926 in the Ukrainian *Bulletin of Reflexology* (in Ukrainian). These studies were not continued, but they contributed to the development of his interest in experimental research.

## The Beginning of a Professional Path

After graduating from the Institute in 1926, Galperin began a medical practice in an evening outpatient clinic and treatment center for drug addicts, treating mainly alcoholics, as well as morphine addicts, cocaine addicts, and others. Galperin suggested that a metabolic disorder is the basis of drug addiction. He translated from German the book *Treatment of drug addictions* and wrote a preface to it, “Outpatient treatment of alcoholics,” which described the organic aspect of drug addiction (Galperin, 1930).

In 1928, Galperin was invited to work in the psychoneurological laboratory, which, like the addiction clinic, was part of the Ukrainian Psychoneurological Institute in Kharkov. Galperin’s publications during those years indicate that he was actively engaged in medical practice, and the results he achieved were the subject of subsequent analysis.

In 1932, the Ukrainian Psychoneurological Academy was formed, as a result of the merger of the Ukrainian Psychoneurological Institute with the Ukrainian Institute of Psychiatry and Psychohygiene, and the creation of a number of new institutions. Around the same time, the People’s Commissariat of Health of Ukraine decided to organize at the Ukrainian Psychoneurological Institute, and then in 1932 at the Psychoneurological Academy, a Division of Psychology, headed by A.N. Leontiev. The researchers in the psychological division of the Academy were A.R. Luria, A.N. Leontiev, P.Ya. Galperin, A.V. Zaporozhets, L.I. Bozhovich, M.S. Lebedinskii, F.A. Khazina, F.V. Bassin, V.I. Asnin, P.I. Zinchenko, V.S. Stepanova, L.P. Odarich, E.I. Artyukh, and G.D. Lukov.^1^ Galperin headed the Section on the General Theory of Psychology, the task of which was to work on the main theoretical problems. In 1934, he wrote, “At present, these tasks are being implemented by defining the subject matter of psychology and, even more precisely, addressing the relationship between the subject matter of psychology and physiology” (Galperin, 1934, p. 35). Galperin already saw the problem of determining the subject matter of psychology as extremely important.

According to Galperin’s recollections, he worked at first with A.R. Luria (Luria soon returned to Moscow), and then joined the Leontiev group. The Kharkov psychologists and graduate students of the Pedagogical Institute and the Research Institute of Pedagogy began to group around Leontiev, and thus the famous Kharkov School of Psychology emerged, with Leontiev as its leader. According to V.P. Zinchenko, there were no ordinary students in this school, and each of the psychologists had their own role to play: A.N. Leontiev — the leader, A.V. Zaporozhets — the conscience, A.R. Luria — the genius, D.B. El’konin — a scientific temperament, P.Ya. Galperin — the teacher. V.P. Zinchenko recalls that people went to Galperin to ask for advice in difficult situations: “Piotr Yakovlevich was … recognized as a teacher. L.I. Bozhovich, A.V. Zaporozhets, P.I. Zinchenko, D.B. El’konin, and many others … said that it was necessary to go consult with the teacher…. The next generations also went to him. … It is hardly necessary to say that Piotr Yakovlevich did not always approve of what he heard. But he was an excellent listener. When, after his sometimes harsh, but kindly and ironically expressed criticism, I went to my immediate teacher A.V. Zaporozhets, discouraged; he consoled me with a smile, saying that he also rarely got to hear compliments from Piotr Yakovlevich” (Zinchenko, 1993, p. 90).

In Galperin’s personal papers there is a document indicating that, at least until February 1936, he remained an associate professor of psychology at the Kharkov Pedagogical Institute. After 1936, Galperin recalls, the Psychology Department was significantly cut back, and he had to transfer to the psychiatric clinic at the Department of Chronic Diseases. From the middle of 1936 until the war began, Galperin was mainly engaged in psychiatry, that is, he worked in his acquired specialty.

Galperin was also actively engaged in teaching in those years. In his autobiography, he writes that his teaching career is officially counted from January 1933, when he began teaching dialectical and historical materialism at the Personnel Institute of the Psychoneurological Academy. He recalls that throughout his life, he paid great attention to teaching, since communication with students contributed to the formation of his own scientific worldview.

## On the Role of Practical Action

In the mid-’30s. Galperin began writing his candidate dissertation on the difference between tools used by humans and animals, and defended it on December 9, 1936 before the Medical Scientific Council of the Psychoneurological Institute. This research was performed under the instruction of the Psychology Department of the Ukrainian Psychoneurological Academy to study “the development of thinking, speech, and practical activity in their interconnection and relationship to other psychological functions at different developmental levels…” (Galperin, 1934, p. 34).

Preserved in Galperin’s personal archive is a version of the dissertation entitled “The difference between human tools and auxiliary aids of animals (experimental psychological research)”; the work is 87 printed pages with illustrations. The title page says: Ukrainian Psychoneurological Academy — President Prof. L.L. Rokhlin, Institute of Experimental Psychoneurology — Directorate: Dr. Z.Yu. Svetnik and Dr. M.O. Kleiman, Section of the General Theory of Psychology — head associate professor P.Ya. Galperin. Later Galperin included the dissertation on the list of his scientific works under a different title, “On the psychological difference between human tools and auxiliary aids of animals.” The official opponents were Professor T.I. Iudin^2^ and Professor M.S. Lebedinskii.^3^

The degree of Candidate of Medical Sciences was awarded by the Higher Attestation Commission only in 1938, since the subject of research chosen by the author was far from medical science. Nevertheless, in 1940 Galperin was confirmed in the academic rank of associate professor in the Department of Pedagogy.

Galperin performed an experimental study of the use of tools by humans and auxiliary aids by animals with the objective, first, to determine the difference between manual operations and tool use, and, second, to clarify the sequence and reasons for the development and change of operations. The use of certain objects by animals, Galperin notes, does credit to their mental abilities and shows that their behavior demonstrates reason. “But this is reason about manual operations; it does not encompass the social usage of objects; it subordinates things to the logic of manual action. It is an instinctive reason … which is limited to discerning the possibility of a mere extension of the arm” (Galperin, 1998, p. 56). The replacement of manual operations by tools leads to a transition of thinking from the path of its biological development, limited by a direct relationship to nature, to a path of development that is “social, mediated by labor and speech and unlimited in its perspective, just as practical human activity is not limited in its perspective and is based on the development of tools” (Galperin, 1998, p. 93). It is not reason that causes a transition from manual operations to tool-based ones, but the opposite: the replacement of manual operations by tools restructures thinking.

Unfortunately, it must be recognized that this work has not yet received its due appreciation, although an abridged form was included in collections of P.Ya. Galperin’s posthumously published works. According to V.P. Zinchenko, “this is the first, without exaggeration, cornerstone of activity theory” (Zinchenko, 2002, p. 122), which “can be confidently called a prolegomenon to psychological activity theory and a test of strength in its experimental substantiation. It convincingly shows the birth of thinking out of practical action and its tasks” (Zinchenko, 2002, p. 121). What V.P. Zinchenko said in the year of Galperin’s 100th jubilee prompts us to turn to one of the first of Galperin’s studies and to work out the internal logic of his scientific views.

## The War Years: Studies on the Restoration of Movement

V.P. Zinchenko asserts that Galperin’s aforementioned studies of children’s tool-mediated activity played an important role in the practical activities of psychologists to restore the movement of those wounded during the Great Patriotic War.

During the war, the Kharkov (Ukrainian) Psychoneurological Institute was turned into a psychoneurological hospital and was evacuated to Tiumen, where Galperin worked as an intern in the neurosurgical department of the hospital from September 1941 through February 1943. Then Galperin moved to the town of Kaurovka, in Sverdlovsk Oblast, where A.N. Leontiev had created a rehabilitation center for restoring movement after injuries. According to Leontiev’s biographers, those working with him also included A.V. Zaporozhets, S.Ia. Rubinstein, T.O. Ginevskaia, Ia.Z. Neverovich, A.G. Komm, V.S. Merlin, among others. Galperin became the head of the medical unit of the rehabilitation hospital. Leontiev’s biography says that he returned from evacuation in the summer of 1943, and Galperin a little later, in October of the same year.

*From then on, from the end of February 1943 until the end of his life, Galperin was on the staff of Moscow University*. His first scientific research at the university was devoted to psychological problems of restoring motor functions after injury.

On February 14–16, 1943, the joint 11th Session of the Ukrainian Psychoneurological Institute and the Second Conference of Neurosurgeons, Neuropathologists, and Psychiatrists of the Ural Military District was held in Tiumen, on the 25th anniversary of the Red Army and the Navy. Galperin gave a report on “The experience of constructing a system of meaningful movements in remedial physical therapy.” He noted that the movement of an affected limb toward an objective target is much more effective than movements performed without any purpose.

Subsequently, in June 1944, the MGU Psychology Department, together with the Institute of Psychology of the Academy of Psychological Sciences and the Clinic of Nervous Diseases of the All-Union Institute of Experimental Medicine, held a special conference on psychophysiological problems of restoring function — motor, sensory, vocal — after military trauma. Galperin, in an article written with T.O. Ginevskaia titled “Dependence of the amount of movement on the psychological nature of the task,” showed that there is a “sharp and unexpected increase in the productivity of movement, crossing boundaries that had seemed insurmountable, due to a change in *the psychological structure of the task*” (Galperin & Ginevskaia, 1947, p. 79).

Although that 1947 publication gives a fairly complete picture of the content of Galperin’s research in evacuation hospitals, it would be unfair not to mention his other printed documents of that time. In 1945, *Uchenye zapiski MGU im. M.V. Lomonosova* published a volume titled “Movement and activity” (a collection of research from the Department of Psychology, edited by S.L. Rubinstein). This volume included an article by A.N. Leontiev, “Psychological study of movement after hand injury,” which describes the results of work in evacuation hospitals. Leontiev stated that “the phenomenon of changing the amount of mobility of the affected parts of the hand, depending on the task being performed, *was first published* by us, *by P.Ya. Galperin*” (emphasis added — M.S.) (Dvizhenie…, 1945, p. 92). He also referred to the Proceedings of the Ukrainian Psychoneurological Institute of 1943, which included Galperin’s work “Psychological factors in remedial physical therapy.”

Also in 1945, Leontiev and A.V. Zaporozhets published the book *Restoration of movement. A psychophysiological study of the restoration of hand functions after injury* (Leontiev & Zaporozhets, 1945), which laid out the results of the work at evacuation hospitals, including by Galperin and Ginevskaia. This study by Galperin and Ginevskaia was used as a textbook, shedding light, among other things, on fundamental issues such as the emergence and development of the activity approach in psychology.

## Moving to Moscow

The Psychology Department within the MGU Philosophy Faculty^4^ was established on October 1, 1942 by S.L. Rubinstein. Until the autumn of 1943, part of the Psychology Department was in evacuation, and A.N. Leontiev was the head of that part of the department. In 1951, Leontiev was named head of the Psychology Department, remaining at this post until 1966, when the Psychology Department was separated from the Philosophy Faculty and became an independent Faculty.

In October 1943, Galperin was approved as an associate professor in the Department of Psychology within the Philosophy Faculty, “for research without a teaching load”; the academic title of associate professor was awarded him in 1946.

Galperin’s theory of the stage-by-stage formation of mental actions and concepts, according to the author himself, developed in the late ’40s to early ’50s as a theoretical solution to a practical school-teaching task: to teach students how to perform arithmetic and grammatical operations, historical and aesthetic analysis, in their minds. However, it can be assumed that the *origin* of some *fundamental* ideas, the formation of *a psychological outlook*, had occurred even earlier. In particular, in 1931, there was a “Discussion on the situation on the psychological front” in Kharkov. Galperin participated and noted the following in his speech: “We must seek *the subject matter of psychology* in the content of such psychological phenomena *as human activity*, and it is with the product of labor (in the broad sense), of human activity, as psychological content, that psychological research should begin (emphasis added –M.S.) (Diskussiia…, 1931, p. 33). Galperin asks about ways to study the content of mental phenomena and explains that since “the psyche *orients* us in fire, water, etc., then the content of mental life must be considered, first of all, in relation to the external world” (emphasis added — M.S.) (Diskussiia…, 1931, p. 33). Thus, the question of the origin and further development of psychology as a science of the orienting nature and function of the psyche is still awaiting comprehensive study.

Galperin presented his theory for the first time in July 1952 at the All-Union Conference on Psychology convened by the Presidium of the RSFSR Academy of Psychological Sciences (a conference on the restructuring of psychological science based on Stalin’s work on linguistics and in light of the decisions of the joint session of the USSR Academy of Sciences and the USSR Academy of Medical Sciences). Galperin was not scheduled to give a report, but he spoke during the debate and set forth a fundamentally different understanding of certain issues of psychology. In his view, there are two lines in psychology: one was that proposed by the author of those remarks with a small number of supporters, and the other was that of the Institute of Psychology of the Academy of Psychological Sciences, with a huge number of supporters. “But … questions of truth are not decided by voting. Truth reflects objective reality, and reality will show itself and be able to stand up for itself ” (Materialy…, 1953, p. 99).

A year later, in July 1953, Galperin delivered a report on “The experience of studying the formation of mental actions in schoolchildren” at a Conference on Questions of Psychology. This was the first detailed and comprehensive presentation by Galperin of his approach to psychology; the report was republished not long ago (Galperin, 2017). In 1955, Galperin took part in two events: the jubilee scientific session in May commemorating the 200th anniversary of the university, and a July conference on psychology.

In 1959, the First Congress of the USSR Society of Psychologists was held in Moscow, at which Galperin gave a report on “The main types of teaching.” The publication in 1959, in the first volume of the academic publication *Psikhologicheskaia nauka v SSSR*, of his foundational article “Development of research on the formation of mental actions” (Galperin, 1959), may be considered a result of the maturing of his theory of the formation of mental actions. This article was the first substantiated presentation of the main theses of the hypothesis (that is how the author describes the system of his ideas) of the formation of mental actions, with a detailed description of the stages of formation and the main parameters of action. By that time, under the leadership of Galperin, experimental research had already begun on the initial formation of the motor skill of writing (N.S. Pantina), geometric concepts (N.F. Tal’zina), the concept of number (V.V. Davydov), grammatical concepts (A.N. Dubrovina, M.Ia. Mikulinskaia), elementary mathematical concepts (L.S. Georgiev), and others. All these studies confirmed Galperin’s assumptions.

Whereas in 1952 Galperin spoke of the small number of psychologists who shared his view on the origin and nature of human consciousness, a few years later ardent opponents emerged, culminating in a discussion that took place in the late ’50s and early ’60s in the pages of the journal *Voprosy psikhologii*. The discussion began with an article in the journal by Galperin, “Mental action as the basis for formation of ideas and images” (Galperin, 1957), which argues that mental action is a psychological phenomenon, while its subject matter is not. In a concluding article in 1960, Galperin “answers” Iu.A. Samarin, A.A. Lyublinskaia, N.A. Menchinskaia, and E.N. Kabanova-Meller, and comes to the conclusion “that various phenomena that are cited to refute the hypothesis actually serve as further confirmation of it” (Galperin, 1960, p. 146).

In the first half of the ’60s, Galperin conducted new research on the theory of the formation of mental actions. In 1962, he headed a group (later a laboratory) on programmed learning, and at MGU, by order of the Ministry of Higher Education of the RSFSR, an interdepartmental group was created to work on issues of programmed learning.

During those years, Galperin took an active part in meetings of Soviet and foreign psychologists: in 1963 at the Second Congress of the USSR Society of Psychologists held in Leningrad (“The experimental formation of attention,” with A.N. Zhdan), in 1964 at the 15th International Congress on Applied Psychology (in Yugoslavia, as part of a delegation), etc.

## Research on the Formation of Mental Actions and Concepts at the Moscow University Psychology Faculty

V.P. Zinchenko drew attention to the fact that Galperin, the last of the brilliant galaxy of L.S. Vygotsky’s school, became a doctor of science and a full professor.

The Academic Council of the Philosophy Faculty of M.V. Lomonosov MGU appealed to the Higher Attestation Commission to allow a defense for the degree of Doctor of Pedagogical Sciences in Psychology based on the set of published works united by the theme “Formation of mental actions and concepts,”^5^ by Associate Professor Galperin Piotr Yakovlevich. Permission was granted.

The defense took place on May 28, 1965 at the MGU Philosophy Faculty, and according to the recollections of psychologists and philosophers who were present, it became an “event” in the scientific life of the university.

Galperin treated the defense with a certain irony, and called the “report … a bureaucratic work, unsuitable for reading, not covering the actual state of affairs and ideas” (from a letter to F.I. Fradkina), and asked “not to place greater demands on it than it deserves” (from a letter to R.G. Natadze) (Galperin, archive).

The transcript of the meeting of the Academic Council on May 28, 1965 (Protokoly …) is preserved in the MGU archive, which makes it possible to reconstruct the procedure of his defense.

In his brief speech, Galperin took up three questions: the psychological structure of human action, psychological mechanisms and laws themselves, and the main method of psychological research.

External comments on Galperin’s published work were prepared by the Psychology Department of Zhdanov Leningrad State University (LGU) and signed by the head of the department, B.G. Anan’ev. Then came the remarks of the first official opponent, B.M. Teplov, and the second official opponent, A.A. Smirnov. The last comments were by G.S. Kostyuk, who, like the two previous speakers, had no fundamental criticisms. The main “criticism” was a complaint that the defense was so late. As V.P. Zinchenko wrote later: “He was coaxed, shamed, and scolded for a long time by friends and students” (Zinchenko, 1993, p. 91). It should be noted that all the speakers emphasized Galperin’s contribution to the development of both general psychology and the psychology of learning.

In conclusion, it should be said that the results of the secret voting were that out of 16 members of the Academic Council present at the meeting, 12 voted “for,” three “against,” and one ballot was invalid.

Galperin’s defense occurred on a memorable day, as Chairman of the Academic Council V.S. Molodtsov said in his speech. It was the same day that the Board of the Ministry of Higher Education decided to create a special Psychology Faculty to replace the Moscow University Psychology Department. Galperin reacted to this event philosophically: on the one hand, he assessed it as very positive, but on the other, with some sadness, since he had assigned a special role to philosophy in the emergence of psychology. Galperin emphasized that he always guided himself by general philosophical methodological problems in psychology, which oriented his work. It should be noted that this idea runs through all of Galperin’s works.

The academic degree of Doctor of Pedagogical^6^ Sciences (MPD Diploma No. 000076) was awarded to P.Ya. Galperin by Decision of the Higher Attestation Commission of January 22, 1966.

After the formation of the Psychology Faculty in 1966, Doctor of Pedagogical Sciences Galperin was appointed associate professor in the Department of General and Applied Psychology.^7^ In February 1967, he was transferred from the position of associate professor to that of professor in the same department. By the Decision of the Higher Attestation Commission of July 12, 1967, P.Ya. Galperin was approved as a professor in the Department of General and Applied Psychology.

In 1966, the 18th International Psychological Congress was held in Moscow, at which Galperin not only delivered a report, “Method, facts and theories in the psychology of the formation of mental actions and concepts,” but was also a co-organizer from the Soviet side, together with J. Piaget and B. Inelder, of the 24th Symposium on “The psychology of concept formation and mental actions” (the symposium’s chairman was J. Bruner). The only discussion between J. Piaget and P.Ya. Galperin took place at this symposium. The difference between the scientific approaches of Piaget and Galperin was not only the basis for the discussion, but also a source that contributed to the development of psychological science. It was Piaget who said at the 18th International Psychological Congress, “We should not be afraid of differences, which encourage us to take the only way to broaden our positions” (cit. by Obukhova, 1995, p. 311).

In 1968, Galperin participated in the Third Congress of the Society of Psychologists of the USSR, which was held in Kiev (“On the characteristics of Type III learning”).

Galperin attended the 19th International Psychological Congress in London in 1969, giving a report on “Learning and mental development (from age 5 to 8).” He gave a report on the same topic to the Fourth All-Union Congress of Soviet Psychologists in Tbilisi.

In November 1970 in Kharkov, on the initiative of the Society of Psychologists of the USSR, the first All-Union Symposium on the Psychology of Memory was held, on “Psychological mechanisms of memory and its patterns in the learning process.” Galperin’s report, “Brief remarks on voluntary and involuntary memory” — following P.I. Zinchenko (1903-1969), to whom he dedicated his speech — drew attention to the role of involuntary memorization in life and learning.

Starting in September 1970, Galperin was the head of the Child Psychology Department; in March 1971 he was elected officially to the post of head of the department.

In 1971–72, there was a discussion about the subject matter of psychology in the pages of the journal *Voprosy psikhologii*. Galperin did not directly participate in the discussion, but archival records have been preserved that shed light on its content.

## “Introduction to Psychology”

Galperin’s principal work, *Introduction to psychology*, was published in 1976 (Galperin, 1976). Archival materials allow us to reliably state that this little book was in preparation for at least 15 years, but was conceived much earlier.

The *Introduction* was written at the beginning of the 1970s — a copy of the manuscript dated 12/27/71 is stored in the archive — but the path to its recognition in the psychological community turned out to be a thorny one. In 1972, the author received a critique from A.N. Leontiev, eight typewritten pages, entitled “Remarks on P.Ya. Galperin’s *Introduction to psychology*.” According to Leontiev, “Much in it remains inarticulated and unclear, and some fundamental methodological problems are *overlooked*” (Galperin, archive). A little over ten years before, Galperin’s hypothesis of stage-by-stage formation of mental actions had been criticized, and now his main work suffered the same fate; one has to wonder at and admire the author’s professional courage: neither then, nor years later, did he abandon his ideas.

A.A. Leontiev and D.A. Leontiev report that A.N. Leontiev and Galperin had a thorough, frank conversation after the *Introduction* was published, since that version had received a great deal of criticism from A.N. Leontiev. “Notes” were written especially for this conversation, page-by-page comments that were included in the volume of A.N. Leontiev’s scientific legacy, *Filosofia psikhologii* [The philosophy of psychology]. In comments to this volume, A.A. Leontiev and D.A. Leontiev sum it up: “P.Ya. Galperin’s theory of stage-by-stage formation, relying in a number of essential provisions on A.N. Leontiev’s activity approach, in fact was a *reduced version* of it, which has proven its practical effectiveness in a number of applied fields, but is hardly suitable for the role of *a general psychological theory*” (emphasis added — M.S.) (Leontiev, 1994).

It should be noted that this view is not shared by all researchers. N.N. Nechaev writes: “I think, however, that here we have something quite the opposite: P.Ya. Galperin’s approach was not a reduction but, on the contrary, a concretization of the activity approach to which de facto, judging from a number of Leontiev’s statements ‘scattered’ through his individual manuscripts and published works, Leontiev was approaching, but which he did not reach” (Nechaev, 2003, p. 59). Nechaev calls Galperin’s approach a concept with an “undisclosed, unexplicated methodological potential” (Nechaev, 2012, p. 37). He urges us to “think about the role that *the theory of stage-by-stage formation of mental actions and concepts* may and should play in the development of the activity approach” (Nechaev, 2012, p. 23). He insists on the possibility of revising the concepts of the activity approach from the standpoint of Galperin’s theory. The author summarizes: this theory “should be considered as a theory of the method of psychological research aimed at reproducing psychological phenomena. This is the essence of the theory of stage-by-stage formation of mental actions, as a modern interpretation of the activity approach. … And we must start from the main point: to begin revising traditional approaches to defining the subject matter of psychology, on the basis of the method of stage-by-stage formation” (Nechaev, 2012, p. 38).

N.F. Tal’zina (2002, 2013) and I.M. Arievich (2002) point to the activity-oriented nature of Galperin’s conception. According to Arievich, “the version of activity theory created by P.Ya. Galperin can serve as a means of understanding many fundamental problems of modern psychology” (Arievich, 2002, p. 50). The author of the present article also conducted a study aimed at finding commonalities and differences in the approaches of A.N. Leontiev and P.Ya. Galperin (Stepanova, 2017). The following conclusion was drawn: there is every reason to consider the theories of A.N. Leontiev and P.Ya. Galperin as *complementary:* they reflect various aspects of the study of activity, its motivational and operational sides, respectively, but neither of them can claim to be a comprehensive analysis of activity.

A comparative analysis of the scientific views of these scientists who worked simultaneously at the Psychology Faculty is the subject of an ongoing special study, so one should not rush to final conclusions. That said, special attention should be paid to those statements by Galperin that are directly related to the issue under discussion.

For many years, the book *Introduction to psychology* was difficult to access: its circulation was only 28,000 copies — which at that time was considered very mo dest — until it was reprinted as part of Galperin’s *Izbrannye psikhologicheskie trudy* [Selected psychological works] (Galperin, 1998). In subsequent years, *Introduction to psychology* went through several more editions. However, it should be added that this small volume requires a lot of work from the reader to comprehend its content.

In 1976, immediately after the publication of *Introduction to psychology*, a review by E.A. Klimov, “A positive development in difficult and acute questions of theory” (Klimov, 1976), appeared in the journal *Voprosy psikhologii*. Publication in the leading and at that time only psychological journal in the Soviet Union serves as proof of the recognition of the significance of Galperin’s book.

## P.Ya. Galperin and L.S. Vygotsky

In 1976, Galperin took an active part in the celebration of the 80-year jubilee of L.S. Vygotsky, on the occasion of which leading Soviet psychologists gave lectures. The period of Vygotsky’s return to psychology had begun. On December 9, 1976 Galperin gave a lecture on “The meaning of L.S. Vygotsky for us today.” It should be added that Galperin considered Vygotsky to be a genius, “a ray of light in the confusion of the psychological crisis” (Galperin, 1981, p. 46).

In 1981, the All-Union Conference took place on “The scientific work of L.S. Vygotsky and the tasks of psychology today,” where Galperin made a presentation on “L.S. Vygotsky’s ideas and the tasks of psychology today.” The main task, in his view, is “… to understand that there is … actually something called mental activity and what in it constitutes the subject matter of psychology” (Galperin, 1981, p. 50). Galperin ended his speech with the following words: “… without overcoming the so-called experimental foundations of classical psychology, it is impossible to build psychology systematically as an objective science. The systematic development of Vygotsky’s ideas is impossible. Because Vygotsky’s ideas are the beginning of a new, non-classical psychology” (Galperin, 1981, p. 50). The use of this quotation is not by chance. I would like to restore historical justice: usually D.B. El’konin is called the discoverer of this reading of L.S. Vygotsky, which does not contradict the actual situation. For example, V.V. Davydov writes, in his “Preface” to El’konin’s *Selected psychological works*, “… D.B. El’konin came to the conclusion that the basic law of human mental development, formulated by Lev Semenovich, … is *the beginning of non-classical psychology*” (El’konin, 1989, p. 21). However, a careful reading of the conference materials (which, unfortunately, have become a bibliographic rarity) shows convincingly that L.S. Vygotsky was simultaneously called the creator of non-classical psychology by both Galperin and El’konin: they were unanimous in their assessment of the cultural-historical psychology created by Vygotsky, which once again shows the unity of the worldview of our leading scientists.

## P.Ya. Galperin’s Scientific School

In 1977, the Fifth All-Union Congress of the Society of Psychologists of the USSR was held in Moscow, at which Galperin gave a lecture on “The problem of activity in Soviet psychology.” The lecture appeared in printed form in 1977, but the materials for it had been prepared long before, since the time that discussion took place about the development of Soviet psychology. In particular, the 1969 so-called “domestic” discussion became well known, and later became the subject of our research (Stepanova, 2017).

The 1960s–’70s turned out to be very productive for Galperin: the hypothesis of the formation of mental actions turned into a developed theory of the origin of specific mental processes and phenomena, confirmed by numerous experimental studies (L.I. Aidarova, G.A. Butkin, M.B. Volovich, I.A. Volodarskaia, L.S. Georgiev, P. Golu, M.M. Gokhlerner, A.N. Zhdan, O.Ia. Kabanova, S.L. Kabyl’nitskaia, I.P. Kaloshina, A.F. Karpova, S.N. Karpova , G. I. Lerner, V.I. Lefevre, N.N. Nechaev, L.F. Obukhova, N.S. Pantina, A.I. Podolskii, Z.A. Reshetova, N.G. Salmina, N.N. Sachko, V.P. Sokhina, N.F. Tal’zina, H.M. Teplenkaia, and others).

In June 1975, the All-Union Conference on “Theoretical problems of managing human cognitive activity” took place in Moscow. The discussion was based on reports in a collection published specially for the beginning of the conference. Galperin prepared an article titled “Management of cognitive activity in terms of perception.”

In 1977, on the initiative of the dean of the Psychology Faculty, A.N. Leontiev, the journal *Vestnik Moskovskogo universiteta. Psikhologiia. Seriia 14* [Bulletin of Moscow University. Psychology. Series 14 (now *Vestnik Moskovskogo universiteta. Seriia 14. Psikhologia*) began appearing. A.N. Leontiev was the editor-in-chief of the journal and P.Ya. Galperin was a member of the editorial board. During Galperin’s lifetime, only one of his articles, “The current state of the theory of the stage-by-stage formation of mental actions” (co-authored with N.F. Tal’zina), was published in the journal (Galperin & Tal’zina, 1979).

In 1979, on Galperin’s 75th birthday, MGU presented him with the title of “Honored Scienti" c Worker of the RSFSR.”

At the Sixth All-Union Congress of the Society of Psychologists, held in Moscow in 1983, Galperin gave a lecture on “The formation of cognitive processes,” which summarized many years of research.

On October 2, 1982, Galperin turned 80 years old. On this occasion, the Rector of Moscow University, Academician A.A. Logunov, issued an order congratulating Galperin on his birthday and expressing gratitude to him. The staff of the Psychology Faculty at Moscow University wrote beautiful words in their congratulatory address: *Your wonderful human qualities, your incomparable charm and captivating wisdom, attract to you people of all ages and professions, because communication with you is a real pleasure. We are proud of being able to work alongside you and to learn from you*.

Galperin remained the head of the department of child psychology (in some documents it is called developmental psychology) until September 1983.

V.P. Zinchenko wrote in his memoirs, “Piotr Yakovlevich ... by nature kept away from leadership, avoided leading positions, ... never tried to qualify for membership in the Academy of Pedagogical Sciences” (Zinchenko, 1993, p. 91). This probably also explains the fact that Galperin did not celebrate anniversaries or birthdays. According to L.F. Obukhova’s memoirs, on his 75th birthday Galperin came together with a small circle of colleagues; photographs have been preserved that help to reproduce the special atmosphere of this almost domestic celebration. The only larger event was the celebration of his 80th birthday and the 55th anniversary of his scientific and pedagogical activity. On September 30, 1982, a meeting of the Academic Council of the Psychology Faculty was held on this occasion. The next day, October 1, a conference was held on “The significance of P.Ya. Galperin’s theory of the stage-by-stage formation of mental actions for the development of psychological science and the improvement of teaching practice.” It was a conference with a large number of participants, which the Psychology Faculty could not accommodate, which is why they held it in a building on the Lenin Hills.

For many years, Galperin had been preparing for publication a work on the relationship between learning and mental development. It was published only in 1985 (Galperin, 1985). A succinct presentation of one of the main problems that was the subject of Galperin’s experimental and theoretical research allows us to identify this edition as having great scientific significance. This brochure was Galperin’s last publication during his lifetime, not counting a short interview with the journal *Voprosy psikhologii* in 1987 on the occasion of his 85th birthday.

P.Ya. Galperin died on March 25, 1988, at the age of 86; his wife, Tamara Izrailevna Meerzon, died six months later. They were buried in Vostriakovskii Cemetery.

## Teaching Psychology

Galperin lectured throughout all his years at Moscow University, He gave a course on general psychology to future philosophers from the beginning of the 1950s to the beginning of the ’80s — more than one generation of psychologists attended these lectures with pleasure and obvious benefit to themselves. True, once — in the 1970/71 academic year — he gave a course in general psychology to psychology students.

Galperin also held seminars on general psychology for psychologists when the lectures were given by A.N. Leontiev, and as V.P. Zinchenko recalled, “we were ensured pluralism and dialogue” (Zinchenko, 1993, p. 91).

For many years, Galperin also taught a year-long course in the history of psychology, which was presented as “a drama of ideas and a drama of people.” In the 1968/69 academic year, Galperin handed over this course to A.N. Zhdan.

Galperin prepared a special course on “The formation of cognitive processes” for students and graduate students of the Psychology Faculty, but did not give it every year.

## P.Ya. Galperin: A Psychologist of the 21st Century

It is commonly known that a person is alive as long as memory of him is alive. The words of V.P. Zinchenko ring true: “It has even become a certainty that psychologists have a memory that is not the weakest of their mental powers. They understand that as long as we remember our teachers, not only they, but also we ourselves, are alive” (Zinchenko, 2021, p. 61).

Among the events of recent years associated with the name of P.Ya. Galperin, the following are particularly worthy of note.

First there was the award of the President of the Russian Federation in the field of education for 1997,^8^ for the creation of the psychological and pedagogical complex known as “Theory and practice of the formation of mental activity,” given to P.Ya. Galperin (posthumously), N.F. Tal’zina, L.I. Aidarova, I.A. Volodarskaia, I.I. Iliasov, L.F. Obukhova, A.I. Podolskii, Z.A. Reshetova, N.G. Salmina, and N.N. Nechaev.

Second, an International Scientific Conference dedicated to the 100th anniversary of the birth of P.Ya. Galperin was held in 2002 at the Psychology Faculty of MGU. The conference, a notable event in scientific life, was attended not only by Galperin’s students and followers from different corners of Russia, but also by foreign colleagues. Similar conferences were timed to coincide with subsequent anniversaries: on the 110th and 115th anniversaries of Galperin’s birth, and a conference is scheduled this year to mark his 120-year jubilee.

Third, the P.Ya. Galperin Lecture Hall was opened at Moscow State University of Psychology and Education (MGPPU) in 2008, on the initiative of L.F. Obukhova, a devoted student and successor in the work of her Teacher. On the 110th anniversary of Galperin’s birth, a lecture hall named after him was also opened at the Psychology Faculty. On the board was written: “Lecture hall named after Piotr Yakovlevich Galperin (1902-1988), an outstanding Soviet psychologist, professor at Moscow University.”

And, finally, Galperin’s works are being republished, and research is continuing on the theory of stage-by-stage formation of mental actions and concepts.

It is now an urgent task to comprehend P.Ya. Galperin’s legacy, which, without any doubt, is not an achievement of the bygone 20th century, but will be one of the present 21st century.

## Footnotes

1 The data are taken from the personal archive of P.Ya. Galperin.

2 Tikhon Ivanovich Iudin (1879–1949) — Russian psychiatrist and historian of Russian psychiatry.

3 Mark Samuilovich Lebedinskii (1894/or1895–1980) — Soviet psychotherapist, in those years a researcher in the psychology sector.

4 A faculty in Russian universities can include several departments *(kafedry)* with a more specific competence — Translator’s note.

5 The defense procedure is described in detail by M.A. Stepanova in “The historical significance of the general psychological theory of P.Ya. Galperin” (Stepanova, 2016).

6 Only in October 1968 was the Decree of the Council of Ministers of the USSR issued “On the inclusion of psychology in the list of branches of science in which academic degrees are awarded.”

7 The structure of the faculty included three departments: general and applied psychology, psychophysiology and neuropsychology, pedagogy and educational psychology; scientific laboratories: psychophysiology of sensations, engineering psychology, neuropsychology, genetic psychology, programmed (guided) learning.

8 Decree of the President of the Russian Federation of October 6, 1998, No. 1200, *Poisk,* No. 41, p. 8.
